# Reducing self-objectification: are dissonance-based methods a possible approach?

**DOI:** 10.1186/2050-2974-1-10

**Published:** 2013-03-19

**Authors:** Carolyn Black Becker, Kaitlin Hill, Rebecca Greif, Hongmei Han, Tiffany Stewart

**Affiliations:** 1 Department of Psychology, Trinity University, One Trinity Place, San Antonio, TX 78212-7200, USA; 2 Rutgers University, GSAPP, New Brunswick, NJ, USA; 3 Pennington Biomedical Research Center, Baton Rouge, LA, USA

**Keywords:** Self-Objectification, Body shame, Cognitive dissonance-based interventions, Eating disorders, Mediation, Sororities

## Abstract

**Background:**

Previous research has documented that self-objectification is associated with numerous negative outcomes including body shame, eating disorder (ED) pathology, and negative affect. This exploratory open study investigated whether or not an evidence-based body image improvement program that targets thin-ideal internalization in university women also reduces self-objectification. A second aim of the study was to determine if previous findings showing that body shame mediated the relationship between self-objectification and eating disorder pathology at a single time point (consistent with self-objectification theory) but did not mediate longitudinally (inconsistent with self-objectification theory) would be replicated in a new sample under novel conditions.

**Methods:**

Ninety-six university women completed a peer-led dissonance-based intervention, along with assessment measures at pre-, post-intervention, 8-week and 8-month follow-up. To address the open trial nature of this study, a planned manipulation check was included to make sure that peer-led dissonance decreased thin-ideal internalization, body dissatisfaction, eating disorder pathology, and negative affect with effect sizes being similar to past randomized controlled trials. We hypothesized that all three subscales of the Objectified Body Consciousness Scale (i.e., self-surveillance, body shame, and appearance control beliefs) would be reduced. In addition, we hypothesized that body shame would mediate the relationship between self-objectification (i.e., self-surveillance) and eating disorder pathology at a both at a single time point and longitudinally.

**Results:**

The planned manipulation check supported the interpretation that peer-led dissonance in this study largely yielded comparable changes to past controlled trials. In terms of changes in dependent variables, results supported all hypotheses with the exception of body shame, which remained unchanged. With regards to the mediation analyses, our first (cross-sectional) hypothesis but not our second (longitudinal) was supported.

**Conclusions:**

Findings provide preliminary support for the use of dissonance interventions in reducing self-surveillance and body control beliefs. Results for body shame and the mediation analyses suggest that greater scrutiny of the body shame construct is warranted.

## Background

Objectification theory is a conceptual framework, rooted in feminist theory, regarding women’s experience of their bodies [1-4]. Objectification theory posits that women’s bodies are regularly objectified (i.e., depicted as sexual “objects to be viewed”) in several cultures, including modern western culture. According to objectification theory, frequent experiences of objectification by others socialize women to engage in self-objectification, whereby a woman internalizes the outside perspective, and subsequently assesses the value of her body based on how others perceive it. Self-objectification can be viewed as adaptive to some degree in that it allows women to anticipate the social ramifications of their appearance [3]. Yet, when discrepancies exist between a female’s body and the external standard she uses to judge her body, negative self-evaluation often ensues [1,4]. Thus, in societies, such as modern western culture, in which the ideal female body type is not only significantly thinner than the average woman but also largely unattainable by virtue of consisting of physically incompatible body attributes (e.g., low body fat and large breasts [4]), self-objectification often results in negative views of oneself.

Research supports the link between self-objectification and a number of negative outcomes for women [2,4,5]. For instance, consistent with objectification theory, habitual self-monitoring of one’s appearance, which characterizes self-objectification, has been associated with increased body shame and appearance anxiety [1]. Furthermore, increased focus on one’s appearance is hypothesized to decrease the mental resources utilized for other activities, subsequently decreasing performance on cognitive tasks, such as math [3]. Perpetual external focus also has been linked to decreased awareness of one’s internal states and ability to obtain peak motivational states [6,7]. Finally, self-objectification appears to be associated with psychological disorders, including unipolar depression, sexual dysfunction, and eating disorders [1,8]. For instance, there is a large body of research supporting a relationship between self-objectification and eating disorders for women ranging in age from adolescence through later adulthood [9-12]. Further, consistent with proposed relationships in objectification theory, body shame appears to at least partially mediate the relationship between self-objectification and measures of eating disorders, albeit at a single time-point [13-15].

Objectification theory has been studied in a number of contexts, including sororities, because some evidence suggests that they may attract women with elevated levels of self-objectification and/or perpetuate or exacerbate this mentality [16,17]. A largely North American tradition, sororities are social institutions for university aged women that aim to meet university women’s need to belong. In a recent study, Rolnik, Engeln-Maddox, and Miller [17] compared levels of self-objectification, body shame, and eating attitudes and behaviors between undergraduate women who participated in sorority recruitment (i.e., “rush”) and those who did not participate at four time points (5 days before recruitment; 4 days into recruitment; bid day; and 1-month after bid day). Recruitment is a sort of courtship process whereby female students determine to which sorority they wish to apply, and sororities decide whom they wish to invite as new members. Rolnik et al. also examined the relationship between BMI and the sorority recruitment process. The authors hypothesized that women who participated in recruitment would show increased levels of self-objectification, body shame, and eating disorder behavior at all time points, as compared to those who did not participate. Further, they predicted that participation in recruitment and joining a sorority would increase self-objectification because recruitment represents a real world example of an objectifying process in that appearance (e.g., conforming to the thin-ideal) is often believed to be highly important during recruitment (see http://sororityrush.webs.com/preparationi.htm). They also predicted that women with higher BMIs would be more likely to drop out of and be less satisfied with recruitment. Results were mixed; findings indicated that women who participated in recruitment had increased levels of self-objectification and eating disorder behaviors and attitudes at all four time points. Evidence did not, however, indicate that recruitment worsened self-objectification. Rolnik et al. also found no difference in body shame between those who did and did not participate in recruitment, although sorority members showed an increase in body shame one month after joining a sorority. BMI negatively correlated with satisfaction with the recruitment process and predicted whether a participant dropped out of recruitment.

Rolnik et al. [17] also investigated proposed relationships in objectification theory (i.e., that self-objectification leads to increased body shame which increases one’s risk for an eating disorder [5]). As noted above, previous cross-sectional research supports objectification theory’s proposed relationships between self-objectification, body shame, and eating disorder pathology with body shame at least partially mediating the relationship between self-objectification and eating disorder pathology when relationships are tested at a single time point [13-15]. Consistent with past research, Rolnik et al. found that body shame mediated the relationship between self-objectification and eating disorder pathology when the model was tested at baseline. Yet, when Rolnik et al. conducted a longitudinal mediation analysis with the same dataset and measures, they found that changes in self-objectification (which was operationalized using the self-surveillance subscale of the Objectified Body Consciousness Scale [2]) did not predict changes in body shame, and that changes in shame did not predict eating disorder attitudes/behavior. In short, a relationship that has been consistently found at a single time point did not hold up when investigated longitudinally. This is an important finding because objectification theory proposes a longitudinal not cross-sectional relationship.

Studying objectification theory under conditions that are likely to increase self-objectification is one approach to investigating this theory. Another approach is to investigate objectification theory and self-objectification under conditions that may reduce self-objectification. For instance, it may be that programs that reduce variables strongly associated with self-objectification (e.g., body dissatisfaction, eating disorder pathology, negative affect [1,2,8-12]) may also reduce self-objectification and offer a novel way to investigate relationships proposed in this theory. Moreover, given the increased recognition about the problematic effects of self-objectification, it is important to begin to study methodologies for reducing self-objectification.

Research supports the use of a peer-led cognitive dissonance-based intervention (PL-DBI) in reducing body dissatisfaction and other eating disorder risk factors in university-based undergraduate communities, like sororities. PL-DBI is supported by both the general research on cognitive dissonance-based approaches [18-24] as well as by specific randomized trials conducted within the sorority context [25-28]. Cognitive dissonance, a well-studied psychological phenomenon, is an uncomfortable psychological state that occurs when actions and beliefs are misaligned [29]. It typically is resolved by altering beliefs to be consistent with one’s actions. Dissonance-based interventions (DBIs) generally aim to induce cognitive dissonance with respect to the thin-ideal standard of female beauty by having participants speak and act against the thin-ideal during a series of small group activities and homework assignments. PL-DBI was started in partnership with an entire community of local sororities at one university, and then expanded in partnership with a national sorority in the United States. This approach also has been implemented in non-sorority settings on select campuses in the United States (e.g., dorms, with intramural trainers; see Becker, Stice, Shaw & Woda [30] for additional detail), as well as in the United Kingdom ( http://www.succeedfoundation.org).

To date, PL-DBI has been implemented with at least one group on over 80 undergraduate campuses in the United States and two campuses in the United Kingdom. Prior randomized controlled research has demonstrated that PL-DBI reduces participants’ levels of thin-ideal internalization, body dissatisfaction, dietary restraint, negative affect and bulimic pathology [27-29], and that sorority peer-leaders who have greater exposure to the program show even greater improvement in these domains [27]. Importantly, PL-DBI has been found to be superior to alternative, credible interventions at post-intervention [28] and at follow-up [26,31]. Further, PL-DBI has generated 14-month within group effect sizes [28] that are comparable to those found for the original version of dissonance prevention in Stice et al.’s [23] randomized controlled trial at 1-year. Follow-up analyses for this study by Stice et al. [21] also found that DBIs can reduce onset of eating disorders at three years.

As previously noted, self-objectification has been linked to body dissatisfaction and disordered eating. Further, self-objectification has been implicated as at least partially mediating the relationship between thin-ideal internalization and body dissatisfaction [32], and research indicates that self-objectification and thin-ideal internalization may have a bidirectional relationship on one another [33]. Thus, it seems plausible that DBIs, which explicitly seek to reduce thin-ideal internalization and have documented success in reducing body dissatisfaction in controlled trials, might decrease levels of self-objectification. DBIs may be particularly salient given that participants are encouraged to collectively battle the thin-ideal as a form of political body activism, an approach that should fit well with attempts to combat objectification. Despite this, DBI’s impact on self-objectification largely has not been explored. To date, one study has examined whether or not a modified version of a DBI reduced self-objectification [33]. In this study, the DBI was specifically altered to include an increased focus on self-objectification and was found to reduce self-objectification. Yet, given that the original DBI (sans the extra focus on self-objectification) has been shown to influence so many factors associated with self-objectification (e.g., eating disorders pathology, body dissatisfaction, thin-ideal internalization, negative affect), it remains possible that the original form of the DBI will impact self-objectification rendering modifications unnecessary.

The first aim of this exploratory open trial was to investigate whether unmodified PL-DBI improves self-objectification. To study self-objectification, we utilized the Objectified Body Consciousness Scale [2], which is composed of three subscales that assess self-objectification and associated constructs: self-surveillance, body shame, and appearance control beliefs. We used this measure to facilitate comparison with Rolnik et al. [17] and to assist with our secondary aim (see below). Self-surveillance is the subscale that typically is used to operationalize self-objectification; thus in the present study, self-surveillance represents self-objectification. Body shame is often viewed as one domain of body dissatisfaction and was important for our secondary aim. Although the appearance control beliefs construct has been identified as less reliable than self-surveillance and body shame [34], given the exploratory nature of this study we chose to include it.

We investigated three hypotheses in the present study and included a manipulation check to address the open nature of this trial. For the manipulation check, we checked to make sure that, as in controlled studies, PL-DBI participants evidenced reductions in thin-ideal internalization, body dissatisfaction, negative affect, and eating disorder pathology. We also benchmarked effects sizes in the present study against effect sizes in previous controlled trials. Previous trials have demonstrated that DBI reduces these risk factors relative to control conditions including alternate interventions (i.e., thus accounting for demand, regression to the mean, placebo). Thus, if effects in the present study are comparable, we have some, albeit not perfect, evidence that reductions in the present trial are not merely a result of demand, regression to the mean etc. We next hypothesized that participants would show improvements in self-surveillance, body shame and appearance control beliefs (Hypothesis 1). If the manipulation check held and hypothesis 1 was supported, we would suggest that self-objectification, body shame, and appearance control beliefs be investigated in future controlled trials.

A secondary aim of this study was to see if the unexpected longitudinal mediation findings from the above-mentioned Rolnik et al. [17] study would be replicated. To our knowledge, Rolnik et al. conducted the first and only published longitudinal mediation analysis regarding self-objectification, body shame, and disordered eating. Thus, we sought to investigate if results would be similar when we investigated the same relationships longitudinally with a different sample that participated in a positive body image program. Like Rolnik et al., we first sought to determine if our sample yielded results consistent with previous cross-sectional research before examining objectification theory longitudinally. If this study does not replicate previous cross-sectional findings, this suggests that any longitudinal findings may be sample specific. However, if the study did replicate standard cross-sectional findings, but did not support a longitudinal relationship, this would represent a bigger challenge to the theory. For our mediation analyses, we first hypothesized that body shame would mediate the relationship between self-objectification and eating disorder pathology at baseline (Hypothesis 3). We next hypothesized that Rolnik et al.’s findings were a random finding, and that we would also find a mediation relationship longitudinally (Hypothesis 4).

## Method

### Participants

Participants were 96 female students at a small southwestern university in the United States who received PL-DBI [35] as part of new sorority member orientation and agreed to participate in a voluntary, associated study. The campus sororities annually and collectively require all new members (i.e., those who have accepted “bids” to join a specific sorority) to complete PL-DBI unless the new members have an excused absence (e.g., class conflict).

Participants’ ages ranged from 18 to 21 years (*M =18.76, SD = 0.77*). The body mass index (BMI) of participants, calculated using self-reported weight and height measurements, ranged from 17.33 to 36.32 (*M = 22.34, SD = 3.38*). We used the NIMH two-question approach to assess ethnicity and race. Twelve percent of respondents endorsed Hispanic/Latino ethnicity, 72% endorsed not Hispanic/Latino and 16% did not respond. In response to the second question, 82% endorsed white, 3% Asian, 2% more than one race, 1% Black, and 12% did not respond. Because the focus of PL-DBI is on prevention and not treatment, as in past studies [25,26,28] we removed four participants from analysis for meeting probable eating disorder status based on the Eating Disorder Examination - Questionnaire (final analyzed sample N =92).

### Procedure

#### Overview

Because the campus sororities require all new members to participate in the annual “Body Image Program,” the “program” (i.e., the PL-DBI program) and “study” (i.e., the assessments only) were separated such that new members could attend the required program and opt out of the voluntary study. As appropriate, the program and overarching study were reviewed and approved by the Institutional Review Board (IRB), Sorority Presidents, Greek Council, and Student Affairs at Trinity University (e.g., Greek Council approved both study and program whereas IRB approved only the study).

Using lists of the new members for each sorority, new members were randomly allocated by undergraduate RAs into groups of 8 to 10, stratified by sorority. Thus, each group had roughly equal representation of members from each sorority. Each group was run by two to four peer-leaders, typically three, drawn from different sororities. At the beginning of the program, held in February, during new member orientation, new sorority members from each of the seven campus sororities attended a brief orientation session. Consenting participants for the voluntary study completed baseline questionnaires using a self-generated ID number to ensure confidentiality and then placed these in a large envelope. All participants were informed that if they chose to participate in the study, they could quit the study at any time without consequence. There was no compensation awarded for participation. To reduce coercion, we also informed all program participants that they could pretend to fill out the questionnaires and return blank questionnaires in the envelope. After completing questionnaires, participants met with their assigned groups to begin the first session. The second session took place exactly one week after the first session. Sessions were designed to last approximately 105 min plus time to complete questionnaires. Consenting participants filled out post-intervention questionnaires after the completion of the second session. Additional questionnaires were completed at 8-week and 8-month follow-up during sororities’ weekly meetings.

#### Participant flow

Of the 92 participants at baseline, 70 (76%) completed questionnaires at post-intervention, 74 (80%) completed questionnaires at 8-week follow-up, and 64 (70%) completed questionnaires at 8-month follow-up.

#### Peer leader recruitment and training

PL-DBI was led by older sorority members. Active sorority members who had previously completed PL-DBI were recruited to be peer-leaders. Interested members were asked to self-screen for body image and eating disorder issues prior to volunteering and were asked not to volunteer if they felt these issues were severe or problematic. We also emphasized that if someone with an eating disorder or significant body image concerns did volunteer, she risked jeopardizing the entire program by making it appear hypocritical. The sororities take great pride in their annual body image program; thus we explicitly asked members to put the greater good of the program above their individual desire to lead a group. This is the same strategy that we have used in our past controlled trials [25-28].

Training for peer-leaders lasted a total of 9 hrs consisting of two 4½ hr sessions held one week apart. Peer-leaders were trained in small groups of 9–12, which were sub-divided into three training teams. Peer-leaders were trained by a doctoral level psychologist (CB) with the assistance of research assistants (RAs). The first session began with an introduction to the program and an overview of the history and impact of the program. Following this introduction, each training team was allotted an equal portion of time to run an abbreviated version of the first session in which they were instructed to follow the script provided in the PL-DBI manual [35]. Each team took turns being the peer-leaders while the other groups acted as mock-participants in the session; this allowed the peer-leaders to experience the session multiple times as well as lead it once.

Immediately after each group completed leading their session, they received supervision that focused on reinforcing group leadership skills and adherence to the manual, corrected any misconceptions or problematic parts of the session, and highlighted teamwork and individual group leader strengths and any observed weaknesses. The second training session was conducted in the exact same manner with each group performing an abbreviated second session while the other groups acted as mock participants.

#### The program

The PL-DBI began with an initial meeting orienting the new members to the program and the study, informing them about the history and structure of the program. Next, we explained the difference between the semi-mandatory program and the voluntary study. Following this, new members who chose to participate then completed consent forms and baseline questionnaires. The program was delivered to each cohort of new members in two 2-hour sessions that were held one week apart.

All groups were run simultaneously. After the initial orientation meeting, the peer-leaders for each group led their new members to the room designated for their sessions. Each room was provided a tape recorder for them to use to record the session; tapes were later used to check adherence. After introductions and icebreakers, the participants discussed the definition and origin of the thin-ideal. They also discussed how various messages from the media, peers, dating partners, families, and culture perpetuate the thin-ideal. Next, participants were given approximately 20 minutes to come up with a list of the costs of pursuing the thin-ideal in their workbooks, after which the group worked together to identify and thoroughly discuss the costs specific to six different areas such as self-esteem and academic costs, which were written on a board by a peer-leader.

Participants then identified examples of times where they encountered pressures to be thin and created verbal challenges for the situations. Each participant shared one of her examples with the whole group. Next, participants received a “mirror homework” assignment, which involved standing in front of a mirror wearing as little clothing as tolerable and writing down all of their positive qualities.

Participants began the second session by reviewing the mirror exercise they completed for homework following the last session, which included stating out loud one physical and emotional quality they liked about themselves. The participants then completed three role-plays with the peer-leaders. In this exercise, each peer-leader adopted a different persona obsessed with pursuing the thin-ideal. Once each character was introduced, participants broke into smaller groups and were given approximately 5 minutes to role-play intervening with each character, as though she was a close friend, with the goal of convincing her not to pursue the thin-ideal. Participants next discussed the role that fat talk plays in perpetuating the thin-ideal and how to challenge it. New members then created a top-10 list of ways to avoid or learn to battle the thin-ideal promoted by the society; this was titled “body activism.” Next, they discussed and listed the ways in which their individual sorority or all of the sororities together could collectively battle thin-ideal; this was referred to as “sorority body activism.” A core aspect of this exercise was to motivate participants to act on the collective sense of political outrage that naturally develops during PL-DBI.

At the end of the final session, members were encouraged to choose one self-affirmation exercise to participate in, either listed in their workbooks or one of their own, to promote more positive talk about, and behavior towards, their bodies. Finally, participants who volunteered for the study completed the post-intervention questionnaires and were dismissed.

### Measures

#### Self-objectification, Body shame and Control beliefs

Self-objectification in the main analyses was operationalized using the14-item version [36] of the Objectified Body Consciousness Scale [2]. The OBCS is composed of three subscales with a total of 14 statements: self-surveillance (four items), body shame (five items), and appearance control beliefs (five items). The self-surveillance subscale is commonly conceptualized as a key indicator of self-objectification [34] and includes statements such as “I often compare how I look with how other people look.” The body shame subscale identifies the degree to which the individual feels shamed by their evaluation relative to the cultural thin ideal, including such statements as “I feel like I must be a bad person when I don’t look as good as I could.” Statements under the appearance control beliefs subscale focus on the extent that the individual feels that they are in control of their appearance using statements such as “I think I could look as good as I wanted to if I worked at it.” For each statement, the participant uses a 7-point Likert scale ranging from *strongly agree* to *strongly disagree* to rate the degree to which each statement corresponds with their own attitude towards their bodies. Responses on the OBCS are averaged for analysis. In college samples, internal consistency for the subscales in the short version of the OBCS (*α =* .66-.89) has been found to be good and comparable to that of the classic version of the OBCS [34]. Likewise, two-week test-retest reliability has been found to be adequate (*r* = .62-.81) and subscale scores for the short version have been found to be highly correlated with the classic version (*r* = .77-.82) [36]. In this study, because of the way we handled reversed scored items for control beliefs, higher scores indicated more favorable appearance control attitudes whereas lower scores represent more positive attitudes with regards to self-surveillance and body shame. In the present sample internal consistency was good for self-surveillance (*α* = .86-92 for all time points) and body shame (*α* = .80-91). Consistent with past research, control beliefs had acceptable though weaker internal consistency (*α* = .53-79). For the mediation analysis, we followed Rolnik et al.’s [17] approach of using the self-surveillance subscale as a sole indicator of self-objectification and the body shame subscale for the construct of body shame.

#### Thin-ideal internalization

Thin-ideal internalization was assessed with the Ideal Body Stereotype Scale-Revised (IBSS-R [37]). This scale consists of 10 items, in which participants endorse how much they agree (1 = *strongly disagree*, 5 = *strongly agree*; scale range: 1–5) with statements such as “thin women are more attractive.” Scores from the items were averaged. In past studies, this scale has demonstrated adequate internal consistency (*α* = .89) and test-retest reliability (*r* = .63) [38,39]. Internal consistency in the present sample was consistent with past research (*α* = .77-.92).

#### Body dissatisfaction

We assessed body dissatisfaction with 9 items from the Satisfaction and Dissatisfaction with Body Parts Scale [40] as done in past dissonance trials by Stice et al [21,23]. Research supports the internal consistency (*α* = .94), 3-week test-retest reliability (*r* = .90), and predictive validity for onset of bulimic symptoms [41]. In this sample, internal consistency was good (*α* = .89-.94).

#### Eating disorder pathology

Eating disorder pathology was measured by taking a composite score of the diagnostic items from the Eating Disorder Examination-Questionnaire (EDE-Q [42]) as previously done in related studies [26,28]. The EDE-Q is a self-report version of the Eating Disorder Examination, which is currently considered the “gold standard” for assessing eating disorder pathology (EDE [43]). The EDE-Q, which has been extensively researched and tested for its psychometric properties [44-46] has been widely used. The 10 diagnostic items assess to what degree participants engaged in eating disordered behaviors over the past 28 days (e.g., “over the past 28 days, how many times have you taken laxatives as a means of controlling your shape or weight?”). Internal consistency for the eating disorder composite was adequate (*α* = .77-.81) in the present sample.

#### Negative affect

We assessed negative affect using the fear, guilt, and sadness subscales from the Positive and Negative Affect Schedule-Revised (PANAS-X [47]). Participants indicated to what degree they had been feeling various emotional states (e.g., nervous, scared, and lonely) over the past few weeks by providing a rating from 1 = *very slightly or not at all* to 5 = *extremely* (scale range: 1–5). Scores from the 17 items were averaged. Prior research with this scale has demonstrated good internal consistency (*α* = .95), convergent validity with affective measures, and predictive validity for bulimic symptom onset [38,47]. In the present study, internal consistency was good (*α* = .90-94).

## Results

### Impact of PL-DBI (Manipulation check and hypothesis 1)

All analyses were conducted on an intent-to-treat basis. Missing data points were handled with PROC mixed procedure with maximum likelihood imputation procedures in SAS. Analyses of variance (ANOVAs) were conducted with time (pre-, post-, 8-week, and 8-month) as the repeated factor to test our main hypothesis that our intervention would improve all dependent measures at post-intervention and follow-up time points. Because the eating disorder pathology composite was skewed, we normalized using a square root transformation. Skewed PANAS data was normalized using a logarithmic transformation. We calculated Cohen’s *d* effect sizes for post-intervention and all follow-ups to facilitate comparisons with previous trials (see Table 1 for means, standard deviations and Cohen’s *d* effect sizes).

**Table 1:** Means and standard deviations for dependent measures

**Measures**	**Baseline *M (SD)***	**Post intervention *M (SD)***	**8-week follow-up *M (SD)***	**8-month follow-up *M (SD)***	**Post intervention *d***	**8 weeks *d***	**8 months *d***
Negative Affect	1.73(.60)	1.50(.49)	1.47(.55)	1.60(.62)	.40*	.38*	.20*
Thin-Ideal Internalization	3.56(.42)	3.05(.72)	3.26(.65)	3.43(.53)	1.2*	.69*	.31
Body Dissatisfaction	3.06(.80)	2.80(.75)	2.98(.76)	2.82(.70)	.35*	.08	.33*
EDE-Q	1.09(.74)	0.87(.63)	0.71(.62)	0.84(.67)	.36*	.47*	.32*
Body Shame	2.82(1.17)	2.78(1.25)	3.03(1.17)	2.84(1.11)	.05	.18	.03
Self-Surveillance	5.01(1.30)	4.55(1.27)	4.44(1.19)	4.70(1.10)	.38*	.43*	.31*
Control Beliefs	3.04(.85)	3.51(.98)	3.54(.75)	3.35(1.03)	.56*	.59*	.38*

Note: Improvement for all measures is indicated by decreasing scores with the exception of Body Control, for which higher scores indicate improvement. * indicates p < .05 for post-hoc t-test comparison with baseline. Cohen’s *d* effect size estimates: .2 = small; .5 = medium; .8 = large [48].

We first investigated whether our four traditional variables (i.e., thin-ideal internalization, body dissatisfaction, eating disorder pathology, and negative affect) showed the typical and expected reductions over time as a manipulation check. We found a statistically significant effect for all four traditional variables (thin-ideal internalization, *F* (3, 214) = 19.95, *p* < .0001; body dissatisfaction, *F* (3, 212) = 5.29, *p* = .0015; eating disorder pathology, *F* (3, 213) = 10.35, *p* < .0001; and negative affect, *F* (3, 214) = 7.79, *p* < .0001). Results of post-hoc comparisons for baseline to each follow-up are presented in Table 1. We then examined baseline to 8-month follow-up within group effect sizes from three previous trials to determine if effect sizes in the present study were comparable. Overall, effects were slightly lower in the present uncontrolled study at 8-months (*d* = .20 - .33) as compared to previous controlled studies (Becker et al. [26]: *d* = .19 - .61; Becker et al. [27]: *d* = .28-.40; 2010: *d* = .30 - .59), even though post hoc analyses at 8-months indicated that most effects were significantly different from baseline. Perhaps ironically, the effect size of the one outcome variable that was not significant at 8-months in the present study (see Table 1), thin-ideal internalization (*d* = .31) was virtually identical to that found in Becker et al. [28] (*d* = .30), where it was significant. Taken as a whole, results from this open trial were reasonably comparable to past research if slightly smaller, and support the interpretation that PL-DBI yielded a similar effect in this trial to past controlled studies.

We next examined our three new variables, each associated with objectification theory. For self-surveillance, we found a statistically significant improvement, *F* (3, 211) = 8.04, *p* < .0001, which was maintained at 8-month follow-up (see Table 1). The magnitude of effect was very similar to our traditional variables. We found a similar pattern when we examined control beliefs, *F* (3, 211) = 10.17, *p* < .0001. Thus, our hypotheses were supported with these two novel variables. Our hypothesis for body shame, however, was not supported. Body shame did not evidence any meaningful change (*d* = .03 at 8 months) during the course of PL-DBI, *F* (3, 211) = 1.78, *p* = .1513.

### Mediation analyses (Hypotheses 2 & 3)

Mediation analysis tests whether the potential mediator variable (a third variable) influences the relationship between the two primary variables of interest. Mediation analyses were conducted to determine if body shame mediated the relationship between self-objectification (operationalized with the self-surveillance subscale) and eating disorder pathology at baseline (Hypothesis 2). We hypothesized that body shame would reduce the effect of self-objectification on eating disorder pathology, or in other words, we hypothesized that the amount of mediation (which is called the indirect effect) would be significant from zero. In order to evaluate if body shame mediated the relationship between self-objectification and eating disorder pathology at baseline, we tested four pathways: (a) self-objectification predicting body shame, (b) self-objectification predicting eating disorder pathology, (c) body shame predicting eating disorder pathology, and (d) body shame mediating the relationship between the other two variables. To facilitate comparison with Rolnik et al. [17], we used similar analyses and performed a series of regression models to test the above pathways as outlined by Baron and Kenny [40]. Next, a bias-corrected accelerated bootstrapping method was used to test if the indirect effect was zero [49].

At baseline, we tested the above pathways with 91 participants. First, it was confirmed that objectification significantly predicted body shame (*b* = .41, *t* = 4.86, *p* < .0001) and self-objectification significantly predicted eating disorder pathology (*b* = .13, *t* = 3.92, *p* = .0002). Next, there was a significant effect of body shame on eating disorder pathology (*b* = .20, *t* = 6.12, *p* < .0001). When controlling for body shame, the effect of self-objectification on eating disorder pathology became non-significant (*b* = .056, *t* = 1.72, *p* = .089). Finally, the indirect effect of self-objectification on eating disorder behavior through body shame was estimated to be .07, with 95% confidence interval from .03 to .12, using a bias-corrected accelerated bootstrap with 5,000 replications [50]. These findings suggest that body shame mediated the relationship between self-objectification and eating disorder pathology at baseline.

**Figure 1:**
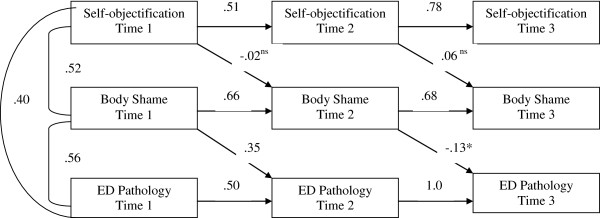
**Cross-lagged panel model examining mediation longitudinally.** Note: ns, not significant at .05 level; *, significant at .01 level; otherwise significant at .001 level.

A cross-lagged panel model was constructed using PROC CALIS in SAS to examine whether the above-mentioned relationship existed longitudinally (Hypothesis 3). We used pre-, post-intervention, and 8-week data and chose not to use eight-month data so that data included in the model were more evenly spaced. In this model, each variable was allowed to predict its own occurrence at subsequent time points; other predictors were examined in addition to those previous effects that had been controlled for, as depicted in Figure 1.

The comparative fit index (CFI) and the standardized root mean square residual (SRMR) were reported. A CFI of .95 or higher and a SRMR of .08 or lower is considered ideal [51]. For the present model, the CFI had a value of .98 and the SRMR had a value of .07, which indicated that the model fit the data well. As shown in the Figure 1, self-objectification at Time 1 predicted self-objectification at Time 2, and the Time 2 value predicted self-objectification at Time 3 (all ps < .001). The same findings held for body shame and eating disorder pathology variables (all ps < .001). However, self-objectification failed to predict body shame when controlling previous occurrence (ps > .05). The results for body shame predicting eating disorder pathology was not consistent over time. Body shame at Time 1 positively predicted eating disorder pathology at Time 2 (p < .001) while body shame at Time 2 negatively predicted eating disorder pathology at Time 3 when previous eating disorder pathology was controlled (p < .01). Since there is no relationship between self-objectification and body shame when controlling for previous occurrence, basic conditions to establish the mediation were not met and the cross-sectional mediation relationship described earlier was not replicated for longitudinal data.

## Discussion

The purpose of the present study was two-fold. First, we sought to determine whether or not self-objectification as measured by the OBCS was reduced in sorority members who participated in a PL-DBI program. Second, we wanted to determine if the mediation findings of Rolnik et al. [17] would replicate under very different circumstances (i.e., during implementation of a positive body image program versus sorority recruitment which could be viewed as increasing body scrutiny).

Regarding the first objective, results indicated that self-surveillance, which is commonly viewed as the OBCS subscale most closely linked with the concept of self-objectification, and control beliefs were both reduced during the course of PL-DBI with effects maintained at 8-month follow-up in new sorority members. Although these findings will need to be replicated in controlled trials, this study provides preliminary evidence that DBIs which reduce thin-ideal internalization and body dissatisfaction may also impact self-objectification, with virtually identical effect sizes in this study. It should be noted that that effect size was relatively small, so additional research will be needed to determine the clinical significance of this finding. Importantly, however, the self-objectification effect size was virtually the same in this study (*d* = .31) at 8-months as in Kroon Van Diest & Perez [33] (*d* = .29) at 5-months. This strongly suggests that the modifications they made to increase the focus on self-objectification were unnecessary.

The current trial was not controlled; thus it could be argued that significant effects were not a result of the program but some other factor such as maturation or testing. Yet, in this trial, effect sizes for body dissatisfaction, thin-ideal internalization, negative affect, and eating disorder pathology were similar to past controlled trials in which dissonance outperformed alternative interventions under the same conditions (peer-led with new sorority members: Becker et al. [25-28]). Moreover, in the Rolnik et al. [17] sorority study, they found no statistically significant changes in self surveillance over time in either the sorority recruitment or control group, suggesting that self surveillance does not reduce merely as an effect of repeated testing over time.

Although participation in PL-DBI was associated with a reduction in self-surveillance and control beliefs, it was not associated with a reduction in body shame. This is a particularly interesting finding in that several constructs hypothesized in the literature to be closely related to body shame (e.g., body dissatisfaction, self-surveillance, eating disorder pathology, thin-ideal internalization) all showed significant and sustained reductions during the course of this study, whereas body shame showed absolutely no change. One possible explanation is that this is simply a random chance finding. This seems unlikely, however, in that Greif, Farris, You, Becker and Wilson [52] found a similar result in a small study of PL-DBI conducted with sororities at a university in the northeast section of the United States. In that study, both the self-surveillance and body control subscales improved, whereas body shame showed no improvement.

Alternatively, it could be that body shame would have naturally increased in this population without the PL-DBI program. Body shame was the one construct that significantly increased in sorority members over the course of Rolnik et al.’s study [17]. Thus, it could be that PL-DBI prevented that increase. This interpretation highlights the need for a follow-up trial that uses a no-intervention control group so that the natural course of body shame is observed.

Yet another explanation is that participants were already fairly low on body shame at baseline with a mean rating of 2.82 which falls between *disagree* and *somewhat disagree* on this scale (sample item: “I would be ashamed for people to know what I really weigh”). This is in contrast to the mean rating of 5.01 on the self-surveillance scale at baseline (sample item: “I often compare how I look with how other people look”), which is equivalent to *somewhat agree*. In other words, this finding could merely indicate a floor effect on this subscale. Interestingly, although baseline scores for self-surveillance are quite similar for the three sorority studies to include this measure (*M* range 5.01-5.29), mean body shame scores for Rolnik et al. [17] and the present study (M range 2.82-2.97) were quite a bit lower than in Basow et al. [16] which reported a mean of 4.03 in sorority members. This provides some support for a possible floor effect.

Another possible interpretation is that the body shame subscale of the OBCS is operationalizing a construct that does not fully map onto the construct originally conceptualized by Fredrickson and Roberts [3] and/or that the relationship between self-objectification, body shame, and eating disorder pathology is not as straightforward as previously thought. These latter interpretations are supported by the mediation findings of this present study and those of Rolnik et al. [17]. To review, Rolnik et al. found that body shame partially mediated the relationship between self-objectification (as measured by self-surveillance) and eating disorder pathology when the relationship was tested at a single time point. This finding was consistent with previous studies (see Tiggemann [53] for review) and consistent with self-objectification theory. Yet, when Rolnik et al. conducted, to our knowledge, the first longitudinal evaluation of the mediating role of body shame, the model was not supported. The present study found almost identical findings. Body shame mediated the relationship between self-objectification and eating disorder pathology at baseline. Yet, when we examined the relationship over time, the mediation model was not supported.

The relative consistency of findings between the present study and Rolnik et al. [17] are striking given that the longitudinal data were collected under quite different conditions. Rolnik et al. collected the data during sorority recruitment, which they argue is a real world situation that could be expected to increase self-objectification. In contrast, we collected our data during the implementation and follow-up of a well-established evidence-based body image improvement program that decreased self-surveillance. The fact that the mediating role of body shame was not supported longitudinally in either study suggests (a) that Rolnik et al.’s findings likely were not spurious, (b) further longitudinal research with other samples is needed, and (c) that researchers may need to rethink the conclusion that body shame as measured by the OBCS can be definitively said to at least partially mediate the relationship between self-objectification and eating disorder pathology in support of self-objectification theory. One interpretation of our study and Rolnik et al.’s is that objectification theory is simply wrong when it comes to the proposed pathway linking self-objectification to eating disorders. This seems a plausible hypothesis but clearly more research is needed before drawing this conclusion, particularly given the potential for floor effects mentioned earlier. It is clear, however, that we cannot assume that cross-sectional support is sufficient for a longitudinal model, and that researchers must begin to study this model longitudinally. Continued cross-sectional research serves little purpose at this point.

Lastly, it may be that body shame is more complex than previously realized. Another way to state this is that factors other than self-objectification may have a significant influence on body shame independent of self-objectification. For instance, Slater and Tiggemann [54] recently found that although time since menarche did not have an influence on self-objectification in adolescent girls, it did have an influence on body shame. More specifically, those girls who began menstruating more recently reported greater body shame. Thus, factors not measured in the present study may have had a significant influence on body shame while not affecting other dependent variables. Even if this is true, however, it suggests that revisions to objectification theory’s proposed eating disorder pathway are likely in need to address this complexity.

This study has a number of limitations. First, as noted above this was an exploratory open trial with respect to the question of the impact of dissonance-based interventions on self-objectification. As such, findings need to be replicated in a controlled trial, ideally with a bigger sample, to address concerns about demand and other treats to internal validity. Although we conducted a manipulation check to make sure effect sizes were comparable to past trials for the traditional variables, controlled research is needed for the new variables. Second, there was some attrition. Although we accounted for that statistically and conducted intent-to-treat analyses, the use of incentives to retain a greater percentage of future samples at follow-up would be beneficial. Third, we were only able to use self-report measures in this study.

## Conclusion

The present investigation provides preliminary evidence that DBIs may impact self-objectification, although further study is needed. This study also adds to a very limited literature suggesting that body shame may not mediate the relationship between self-objectification and eating disorder pathology longitudinally, despite significant evidence that the proposed mediating model is supported at a single time point. This, combined with the finding that body shame did not reduce over the course of the PL-DBI program while highly related constructs did improve, suggests that the body shame construct and the subscale of the OBCS deserve further critical scrutiny as does objectification theory and the proposed pathway from self-objectification to eating disorders.

## Competing interests

Dr.’s Carolyn Black Becker and Rebecca Greif have received compensation in the past for time spent training people in the use of dissonance-based interventions. Dr. Becker is also co-director of the Body Project Collaborative, LLC which was formed to facilitate dissemination of dissonance-based interventions.

## Authors’ contributions

CB, RG, KH, and TS conceptualized this paper. CB and KH collected the original data for the paper. CB, RG, KH, and TS all contributed to the writing, editing, formatting of the paper. KH managed and organized the database. HH conducted the statistical analyses and reviewed write up of analyses. HH, CB, and TS reviewed and interpreted the results. All authors read and viewed the final version of this manuscript.
